# Specific Deletion of LDL Receptor-Related Protein on Macrophages Has Skewed *In Vivo* Effects on Cytokine Production by Invariant Natural Killer T Cells

**DOI:** 10.1371/journal.pone.0102236

**Published:** 2014-07-22

**Authors:** Roman Covarrubias, Ashley J. Wilhelm, Amy S. Major

**Affiliations:** 1 Department of Pathology, Microbiology and Immunology, Vanderbilt University Medical Center, Nashville, Tennessee, United States of America; 2 Department of Medicine, Division of Cardiovascular Medicine, Vanderbilt University Medical Center, Nashville, Tennessee, United States of America; Institut Pasteur, France

## Abstract

Expression of molecules involved in lipid homeostasis such as the low density lipoprotein receptor (LDLr) on antigen presenting cells (APCs) has been shown to enhance invariant natural killer T (iNKT) cell function. However, the contribution to iNKT cell activation by other lipoprotein receptors with shared structural and ligand binding properties to the LDLr has not been described. In this study, we investigated whether a structurally related receptor to the LDLr, known as LDL receptor-related protein (LRP), plays a role in iNKT cell activation. We found that, unlike the LDLr which is highly expressed on all immune cells, the LRP was preferentially expressed at high levels on F4/80^+^ macrophages (MΦ). We also show that CD169^+^ MΦs, known to present antigen to iNKT cells, exhibited increased expression of LRP compared to CD169^-^ MΦs. To test the contribution of MΦ LRP to iNKT cell activation we used a mouse model of MΦ LRP conditional knockout (LRP-cKO). LRP-cKO MΦs pulsed with glycolipid alpha-galactosylceramide (αGC) elicited normal IL-2 secretion by iNKT hybridoma and *in vivo* challenge of LRP-cKO mice led to normal IFN-γ, but blunted IL-4 response in both serum and intracellular expression by iNKT cells. Flow cytometric analyses show similar levels of MHC class-I like molecule CD1d on LRP-cKO MΦs and normal glycolipid uptake. Survey of the iNKT cell compartment in LRP-cKO mice revealed intact numbers and percentages and no homeostatic disruption as evidenced by the absence of programmed death-1 and Ly-49 surface receptors. Mixed bone marrow chimeras showed that the inability iNKT cells to make IL-4 is cell extrinsic and can be rescued in the presence of wild type APCs. Collectively, these data demonstrate that, although MΦ LRP may not be necessary for IFN-γ responses, it can contribute to iNKT cell activation by enhancing early IL-4 secretion.

## Introduction

The activation of invariant natural killer T (iNKT) cells has been shown to impact disease progression in mouse models of human disease such as multiple sclerosis [1], [2], atherosclerosis [3], [4] systemic lupus erythemathosus [5], cancer [6] and pathogenic infection [7]. In humans and mice, iNKT cells rearrange their T cell receptor (TCR) to express Vα24-Jα18 and Vα14-Jα18, respectively [8]. This allows iNKT cells from both species to recognize similar glycolipid antigens and elicit potent immune responses. Unlike conventional adaptive immunity mediated by CD4^+^ and CD8^+^ T cells that requires 4–6 days to initiate, the activation of iNKT cells occurs quickly after *in vivo* glycolipid challenge leading to a rapid secretion of effector cytokines including IL-4, IL-12 and IFN-γ which can be detected as early as 90 minutes following activation [9]. Additionally, iNKT cell responses are known to enhance the initial phase of immunity by increasing NK, B and T cell activation. This effect, known as immune transactivation, is thought to link innate immunity to adaptive immune responses [10] capable of fending off infection [11] and priming effector CD4^+^ and CD8^+^ T cells [12]. Therefore, modulation of iNKT cell responses is a potential therapeutic approach for cancer, autoimmunity and chronic inflammatory disorders.

Although research has vastly increased our knowledge of iNKT cell function, less is known about the cellular components in antigen presenting cells (APCs) that present glycolipid ligands to activate iNKT cells. Studies focused on understanding iNKT cell immunity have shown that proteins classically involved in lipoprotein metabolism, such as the microsomal triglyceride transfer protein (MTP) [13], low density lipoprotein receptor (LDLr) [14], scavenger receptors (SRs) [15], and cholesterol membrane transporters [16] can modulate iNKT cell homeostasis and activation. The mechanisms by which MTP, LDLr and SRs on APCs modulate iNKT cell activation are unknown, but data suggest a critical role in glycolipid uptake and presentation. MTP has been shown to be essential in the loading of endogenous lipids into the hydrophobic pocket of CD1d [17] serving to stabilize this molecule for appropriate expression. MTP-deficient APCs lack CD1d expression on the cell surface and iNKT cells fail to develop in fetal thymus organ culture when treated with MTP inhibitors [18]. On the other hand the LDLr binds to apoE present on the surface of many lipoproteins which can function as vessels for glycolipid transport. Targeting glycolipid uptake via the LDLr in APCs enhances CD1d-mediated antigen presentation and leads to increased iNKT cell activation [14], [19]. This shows that molecular pathways related to lipoprotein homeostasis and metabolism are closely linked to the modulation of iNKT cell function.

A similar protein to LDLr initially described in lipoprotein metabolism, but later found to bind approximately 30 different ligands, is the LDL receptor-related protein (LRP, also referred to as CD91) [20]. In mice, the LRP was initially discovered in hepatocytes [21], but is currently thought to be expressed in most cells of the body, including many immune cells [22]–[26]. The expression of LRP on APCs has been shown to enhance the adaptive immune response by facilitating antigen uptake [27]–[29].^.^ LRP can modulate innate immune responses by binding pseudomonas exotoxin A [30], rhinovirus particles [31] and collectins [32]. For iNKT cell activation, the LRP may be important by binding apoE-containing lipoproteins [33], facilitating processing of the lipid transport proteins called saposins (necessary for CD1d loading) [34] and/or by interacting with the endoplasmic chaperone protein calreticulin on the cell's membrane leading to phagocytosis of opsonized pathogens and apoptotic bodies [35]. Given these characteristics of LRP and the fact that it can actively recycle through endocytic comparments [36], we hypothesized that this surface receptor plays an active role in glycolipid antigen presentation and subsequent activation of iNKT cells. In this study, we demonstrate that LRP is highly expressed in specialized macrophages (MΦs) capable of iNKT cell activation. We also show that MΦ LRP can modulate iNKT activation *in vivo* and is necessary for IL-4, but not IFN-γ secretion.

## Materials and Methods

### Animals

Lysozyme M **(**LyzM)-Cre^+/-^,LRP^flox/flox^ (hereafter referred to as LRP-cKO) mice were a kind gift from Dr. Sergio Fazio (Department of Medicine, Vanderbilt University Medical Center) and have been described previously [37]. Briefly, mice harboring loxP sites flanking the LRP gene [38] were crossed with mice expressing cre-recombinase under the control of macrophage LyzM promoter [39]. Littermate LyzM-Cre^-/-^, LRP^fl/fl^ were used as wild type (hereafter referred to as WT) controls. For some experiments, C57Bl/6 mice were used as indicated. B6.PL-Thy1<a>/CyJ mice were obtained from the Jackson Laboratory. All mice were on the C57BL/6 background. Mice were maintained in the Vanderbilt University animal care facility and had access to food and water *ad libitum*. All procedures were approved by Vanderbilt University Medical Center's Institutional Animal Care and Use Committee.

### Cell isolation

Isolation of leukocytes from liver and spleen has been described previously [40]. In short, spleens were digested with 1 mg/mL collagenase type II (Sigma) in Hank's balanced salt solution (HBSS, Mediatech). Digested spleens were passed through a 70 µm cell strainer and red blood cells lysed by osmotic shock. Livers were perfused with cold PBS, digested with collagenase type II and pressed through a 70 µm cell strainer. Hepatic leukocytes were then isolated from the interface of a 40/60% Percoll gradient (GE Healthcare, Piscatway, NJ). Peritoneal macrophages were collected in PBS 3 days after peritoneal injection of 3% thioglycollate (Fluka).

### Flow Cytometry

Single-cell suspensions of mononuclear leukocytes were blocked for 15 minutes at RT in a 1∶50 dilution of Fc receptor block (BD Biosciences) in FACS buffer (1X HBSS, 1% BSA, 4.1 mM sodium bicarbonate, and 3 mM sodium azide). The following fluorescently labeled antibodies were diluted 1∶100 in FACS buffer and incubated with cells for 45 minutes at 4°C: CD11b-V450, Thy1.1-V450 (1∶300 dilution), CD69-FITC, CD1d-PE, CD4-PE, TCRβ-PE, CD8-PerCP, CD45-PerCP, Thy1.2-PeCy7, CD11c-APC and CD45R-APC-cy7 (all from BD Pharmingen), NK1.1-PerCP, F4/80-PeCy7 (from eBiosciences) and Siglec-1-PE (Biolegend) LY49C/F/H/I (clone 14B11, from BD Biosciences). To stain iNKT cells, α–galactosylceramide-CD1d tetramers- APC (NIH tetramer facility) were used. iNKT cells were defined as TCRβ^+^B220^-^ TCRβ^int^ tetramer^+^. To stain LRP for flow cytometry, we fluorescently labeled 5A6 LRP clone (gift from Dr. Dudley Strickland, University of Maryland) with APEX Alexa Fluor-488 antibody labeling kit (Invitrogen) according to manufacturer's protocol. Conjugated 5A6-Alexa Fluor-488 antibody was titrated to determine optimal binding dilution. In order to stain total levels of LRP (surface and intracellular), cells were stained for surface markers prior to staining with LRP antibody. Surface labeled cells were then fixed and permeabilized with Cytofix/Cytoperm (BD Pharmingen) reagents according to manufacturer's protocol. Mouse IgG2b-Alexa Fluor 488 as the matched isotype control (BD Biosciences). Labeled cells were analyzed on a MACSquant seven-color flow cytometer (Miltenyi Biotec) and data analyzed with FlowJo software (Tree Star).

### Enzyme-linked Immunosorbent Assay

Mouse IL-2, IL-4, IFN-γ and IgE were measured by standard sandwich ELISA according to the manufacturer's protocol (BD Pharmingen).

### Measurement of *In Vitro* and *In Vivo* Responses to αGC

*S*plenocytes were plated at 2.5×10^5^ cells/well in RPMI (Hyclone) media containing 10% FBS (Sigma), penicillin-streptomycin with 50 µmol/L L-glutamine (Gibco) and 50 µmol/L β-2-mercaptoethanol (Sigma) with the indicated concentrations of αGC. Supernatants were collected after 72 hours of culture and cytokine levels determined by ELISA. For peritoneal MΦs, 1.0×10^5^ cells/well were incubated with 1.0×10^5^ of murine iNKT hybridoma DN32.D3 (from Dr. Albert Bendelac, University of Chicago, described in [41]). Supernatants were collected 48 hours after culture and IL-2 measured by sandwich ELISA. For *in vivo* measurements, 4 µg, 1 µg or 0.5 µg αGC reconstituted in vehicle buffer (0.5% polysorbate) was i.p. injected in a total volume of 200 µl. An equal volume of vehicle was injected in to control mice. At the specified times following injection, plasma was collected and splenocytes were isolated, stained and analyzed by flow cytometry.

### Pulse of Peritoneal MΦs with BODIPY-αGC

Peritoneal MΦs (pMΦs) were obtained as described previously. A total of 5×10^5^ cells/tube were incubated with 1 µg/mL BODIPY-αGC (gift from Dr. Paul Savage, Brigham Young University) and harvested at the time points noted in the text. For the pulse-chase experiment, pMΦs were preincubated with αGC for 4 hours. After this time, cells were washed an incubated with BODIPY-αGC.

### Generation of mixed bone marrow chimeras

WT Recipient mice were irradiated with 950 rads using a cesium source and bone marrow from donor mice was transplanted via retro-orbital injection with a 1∶1 mixture (2×10^6^ total cells) from LysMcre^+/-^, LRP^flox/flox^ (Thy1.2) and C57BL/6 (Thy1.1) mice. Recipient mice were maintained on sterile water with sulfamethaxazole-trimetroprim for 3 weeks. Chimeras were analyzed 4 weeks after transplant.

### Immunization with αGC and induction of Immunoglobulin E (IgE)

Mice were injected with 4 µg/mouse of αGC or vehicle buffer (0.5% polysorbate). Serum was collected prior to injection and also 6 days after immunization to measure IgE levels.

### Endogenous lipid presentation assay

We used DN32.D3 hybridoma (1.0×10^5^/well) with thymocytes (1.0×10^6^) in 200 µL/well RPMI for 48 h, supernatants were collected and IL-2 levels measured by ELISA.

### Statistical Analyses

Statistical analyses were conducted using PRISM V.5.0 software (GraphPad, La Jolla California, USA). For direct comparison between two groups, an unpaired Student's *t*-test was used and for comparisons made between three or more groups a one-way analysis of variance (ANOVA) was performed. Values are expressed as mean ± standard error of the mean unless otherwise noted. A *p* value <0.05 was considered statistically significant.

## Results

### LRP is differentially expressed on immune cells

**Figure 1:**
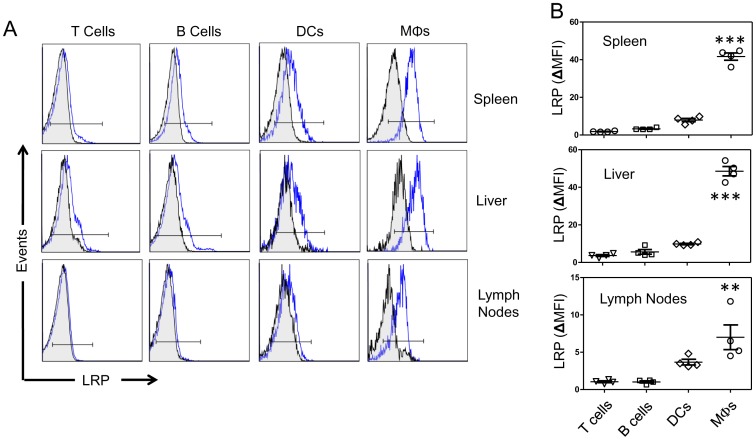
LRP expression in T cells, B cells, DCs and MΦs. **(A)** Representative histograms for LRP (blue line) versus isotype control (black line) staining in spleen (top row) liver (middle row) and lymph nodes (bottom row). Mononuclear lymphocytes from from 8-to-12 week old WT mice (n = 4) were stained with fluorochrome-conjugated antibodies for T cells (TCRβ), B cells (B220), DCs (CD11c) and MΦs (F4/80). **(B)** Graphs showing quantification of mean fluorescent intensity (MFI  = LRP Antibody- isotype antibody) of LRP on lineage-specific populations. Symbols represent individual mice. Bars represent mean and standard error. *** denotes *p*<0.0001 compared to T cells, B cells and DCs. ^**^denotes *p*<0.001 compared to T cells and B cells. *P* value was determined by a one-way ANOVA with a Bonferroni post-test. Histograms are representative of three separate experiments with 4 mice each. Scatter plots show individual mice from one representative experiment of three separate experiments.

We measured LRP expression on immune cells isolated from tissues in which iNKT cells are known to reside and encounter antigen. We collected spleen, liver and lymph nodes from C57Bl/6 mice and stained with fluorescently labeled antibody (5A6) specific for the 85 KDa subunit of LRP [42]. Flow cytometric analysis demonstrated that F4/80^+^ splenic MΦs express the highest levels of LRP (ΔMFI  = 44.7±2.4) compared to T cells (ΔMFI  = 1.6±0.4), B cells (ΔMFI  = 10.4±.35) and DCs (ΔMFI  = 15.1±0.7) (Figure 1A, B, top row). Similar LRP expression profiles were observed on immune cells isolated from the liver and lymph nodes. (Figure 1 A, B middle and bottom rows).

### CD169^+^ MΦs express high levels of LRP

**Figure 2:**
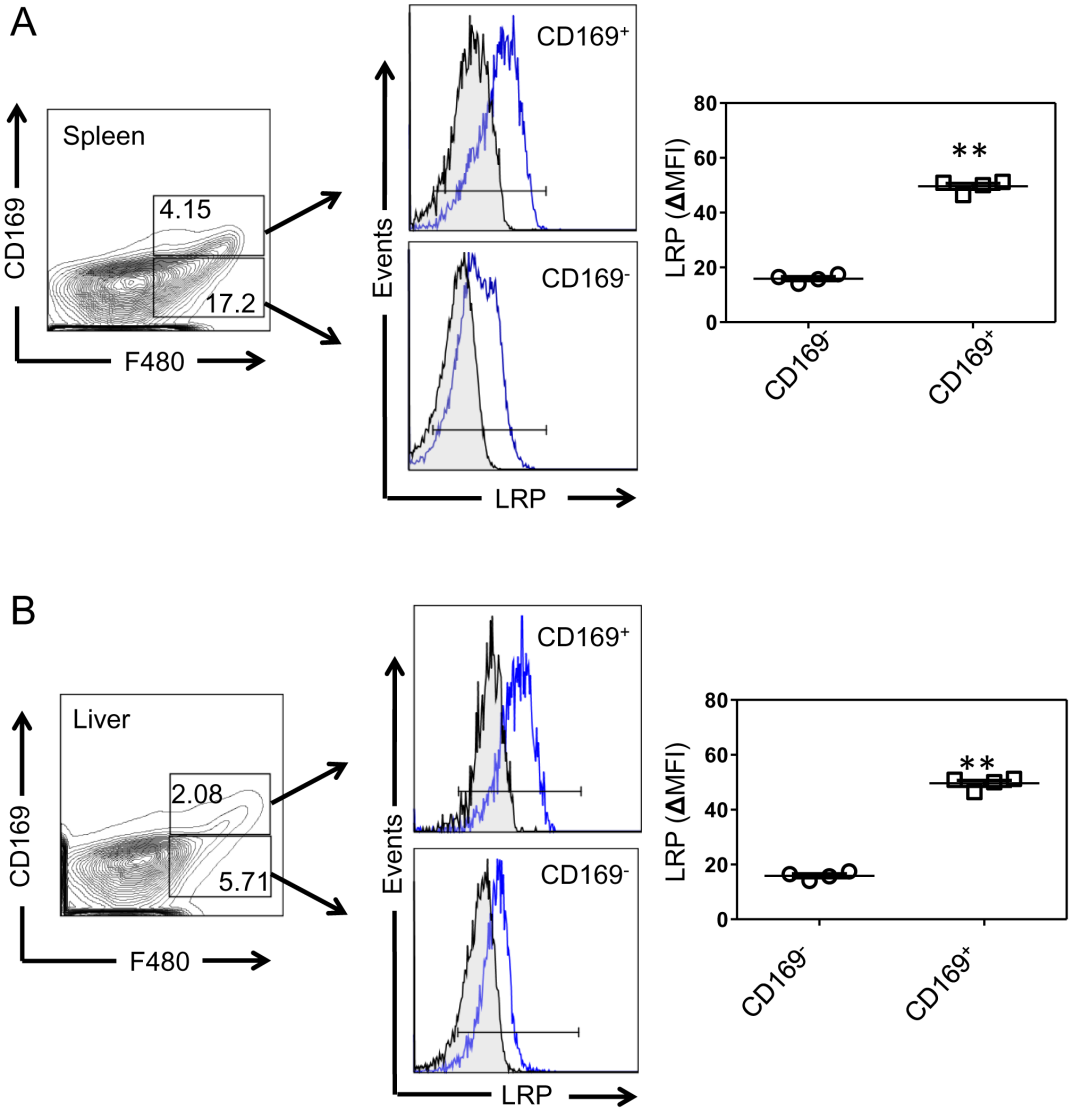
LRP expression in CD169^+^ MΦs. **(A)** Spleen and **(B)** liver mononuclear lymphocytes from 8-to-12 week old WT mice (n = 4) were stained with fluorochrome-conjugated antibodies for MΦs (F4/80) and CD169. Left panels show dot plots for F4/80 and CD169 and middle panels show representative histograms of LRP expression (blue line) versus isotype control (black line). Graphs show quantification of mean fluorescent intensity (MFI  = LRP antibody- isotype antibody). Bars represent mean and standard error. ^**^denotes p<0.001. *P*-value determined by Student's *t*-test. Histograms are from one representative experiment of three separate experiments. Scatter plots show individual animals from one representative experiment of three.

Recent reports have shown that MΦs expressing sialic acid binding immunoglobulin-like lectin 1 (Siglec-1, also known as CD169) can mediate potent activation of iNKT cells in secondary lymphoid tissues [43]. CD169^+^ MΦs can cross-present tumor antigens [44] and take up liposomal particles conjugated to CD169 ligands [45]. One such study demonstrated that αGC conjugated to CD169 ligands elicited an iNKT cell response two orders of magnitude higher that administration of free αGC [46]. Interestingly, we found significantly higher LRP expression levels on F4/80^+^CD169^+^ MΦs compared to F4/80^+^CD169^-^ MΦs in spleen (Figure 2A) and liver of C57Bl/6 mice (Figure 2B). These data demonstrate that LRP is highly expressed on MΦs known to elicit iNKT cell responses.

### Conditional knockout leads to deletion of LRP on MΦs

**Figure 3:**
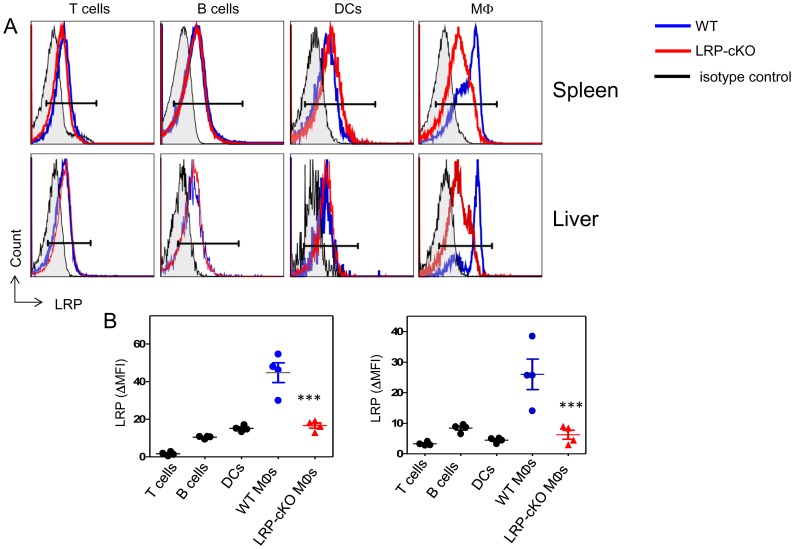
LRP-cKO mice exhibit knockout of LRP in MΦs. **(A)** Spleen and liver mononuclear cells from 8-to-12 week old WT and LRP-cKO (n = 4) were stained with fluorochrome-conjugated antibodies for T cells (TCRβ), B cells (B220), DCs (CD11c) and MΦs (F4/80). **(B)** Graphs show quantification of mean fluorescent intensity (MFI  =  LRP antibody MFI- isotype antibody MFI) of LRP. Each symbol represents an individual mouse from one representative experiment of three separate experiments. Bars represent mean and standard error. *** denotes *p*<0.0001 and ** denotes *p*<0.001 when comparing WT MΦs to LRP-cKO MΦs, *p* value was determined by Student's *t* test.

In order to test MΦ LRP function in iNKT cell activation, we used mice harboring a MΦ knockout of LRP [37]. In this mouse model, LRP^flox/flox^ mice were crossed with LyzM-Cre mice, which have cre recombinase under the control of the lysozyme M promoter. Flow cytometry staining of splenic and liver MΦs (Figure 3A and 3B) shows that, compared to WT, LRP-cKO mice have decreased LRP expression on splenic and hepatic MΦs (71% and 68%, respectively). As expected, other immune cells in LRP-cKO mice such as T cells, B cells and DCs had no reduction of LRP expression, verifying MΦ specificity. Further analysis of LRP-cKO MΦs subpopulations showed that CD169^+^ MΦs have a 53% reduction in LRP expression (Figure 2A-B). Taken together, these data show that LRP-cKO mice have a deletion of LRP on MΦ, including specialized CD169^+^ that are capable of presenting antigens to iNKT cells.

### LRP deficiency on MΦdoes not alter CD1d expression or iNKT cell homeostasis

We asked whether LRP deletion in MΦs leads to changes in CD1d expression or iNKT cell homeostasis. LRP deletion had no effect on CD1d expression either on spleen MΦs, liver MΦs, or double positive (CD4^+^CD8^+^) thymocytes (Figure S1A). Co-cultures of WT or LRP-cKO thymocytes with iNKT hybridoma (DN32.D3) led to similar levels of IL-2 secretion (Figure S1B), showing that LRP-cKO thymocytes have no defects in presentation of endogenous glycolipid ligands. Analysis of thymic cells demonstrates normal expression of development markers in LRP-cKO iNKT cells when compared to WT (Figure S2). Additionally, iNKT cells, isolated from spleen and liver were present at normal frequencies (Figure S3A-C) and expressed normal levels of the prototypic markers of homeostasis and activation (Figure S3D and S3E). Finally, *in vitro* uptake of BODIPY-labeled αGC was not affected by LRP deficiency on MΦs as measured by flow cytometry (Figure S4A and S4B). However, a pulse-chase experiment where unlabeled αGC was used to pulse perotineal macrophages (pMΦs) prior to chase with BODIPY-αGC showed a faster turnover of LRP-cKO pMΦs as BODIPY-αGC was higher than WT pMΦs (Figure S4C). This difference is observed as early as 4 hours but reaches statistical significance only at 8 and 16 hours. These data suggests that LRP deletion in MΦs does not affect uptake of glycolipids but can alter cellular glycolipid turnover.

### LRP deficiency on MΦs does not affect iNKT activation *in vitro*

**Figure 4:**
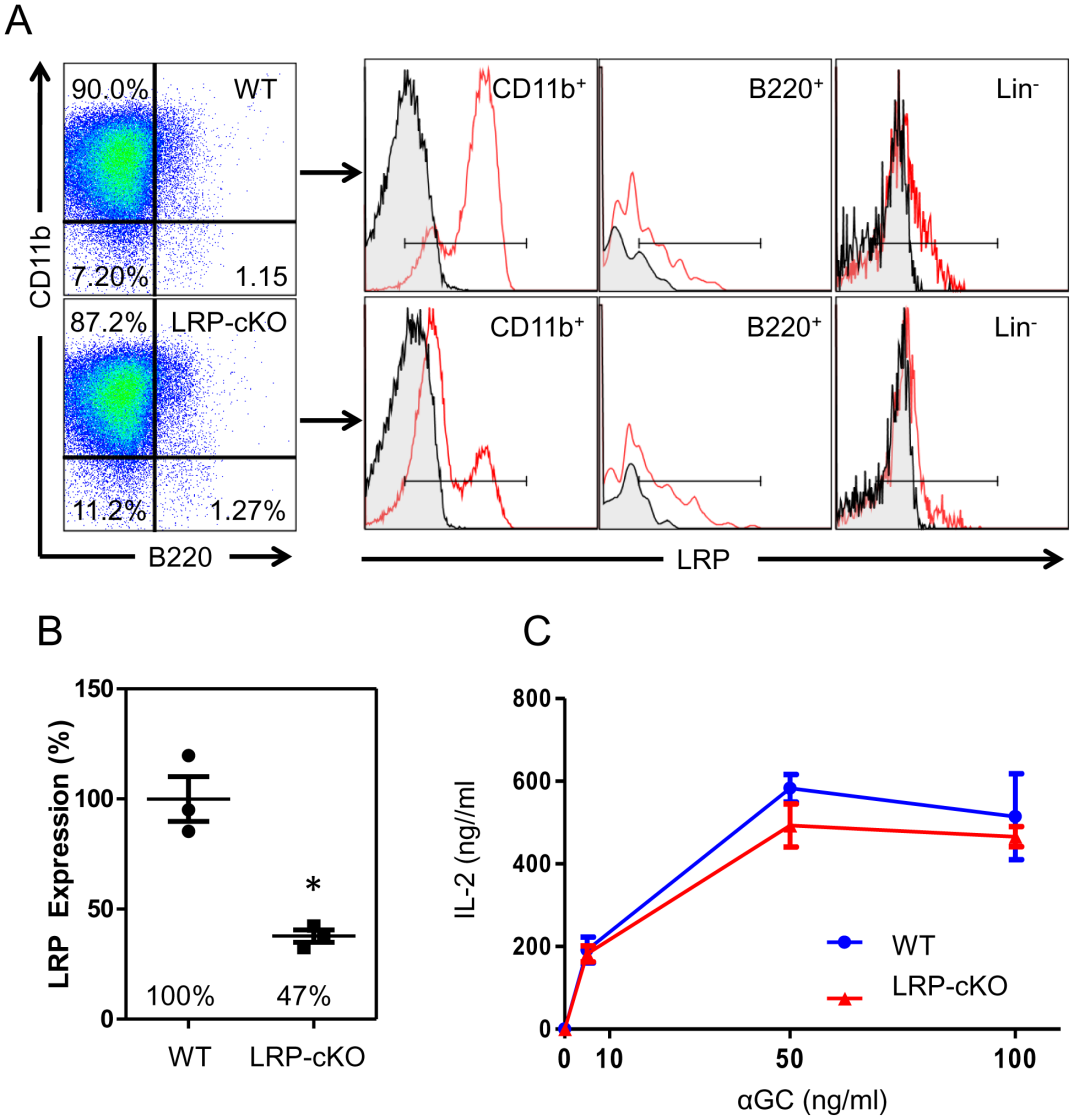
LRP deletion in pMΦs does not affect IL-2 secretion by iNKT cell hybridomas. **(A)** Left panel shows representative flow cytometry dotplots of WT and LRP-cKO macrophages along with histograms showing LRP expression in macrophages (CD11b^+^) B cells (B220^+^) and lineage negative cells from the peritoneal cavity. **(B)** Quantification of LRP expression in pMΦs from WT (n = 3) and LRP-cKO (n = 3) mice. Each data point represents an individual mouse and results are from one representative experiment of three separate experiments **(C)** WT (n = 3) and LRP-cKO (n = 3) pMΦs incubated with iNKT cell hybridomas for 48 hours. Each sample well was done in triplicates and IL-2 levels were determined by ELISA. Results are from one representative experiment of three separate experiments. Shown bars represent mean and standard error.

Although we did not see changes in uptake of αGC by LRP-deficient MΦs, LRP is also important for cell signaling. Therefore, to determine whether decreased LRP expression modulates iNKT cell activation, we first harvested thioglycollate-elicited pMΦs from WT and LRP-cKO mice and determined their LRP expression. Similar to previous reports [37], we found a 63% decrease in LRP expression on LRP-cKO pMΦs when compared to WT (Figure 4A and B). To measure iNKT cell activation, pMΦs were pulsed with αGC and incubated with iNKT cell hybridoma, DN32.D3, and secreted IL-2 was measured in 48 hour culture supernatants by ELISA. We observed a slight but reproducible decrease in IL-2 when iNKT cells were incubated with LRP-cKO pMΦs (Figure 4C). Unfortunately, this did not reach statistical significance. *In vitro* incubation of freshly isolated splenocytes from WT or LRP-cKO mice with αGC for 24, 48, 72 hours likewise showed no statistically significant difference in IFN-γ or IL-4 levels in culture supernatants (Figure S5), but a slight reduction in IL-4 at 48 hours of culture. These data suggest that *in vitro* activation of iNKT cells by MΦs is not dependent of LRP expression.

### LRP deficiency alters iNKT cell secretion of IL-4 but not IFN-γ *in vivo*

**Figure 5:**
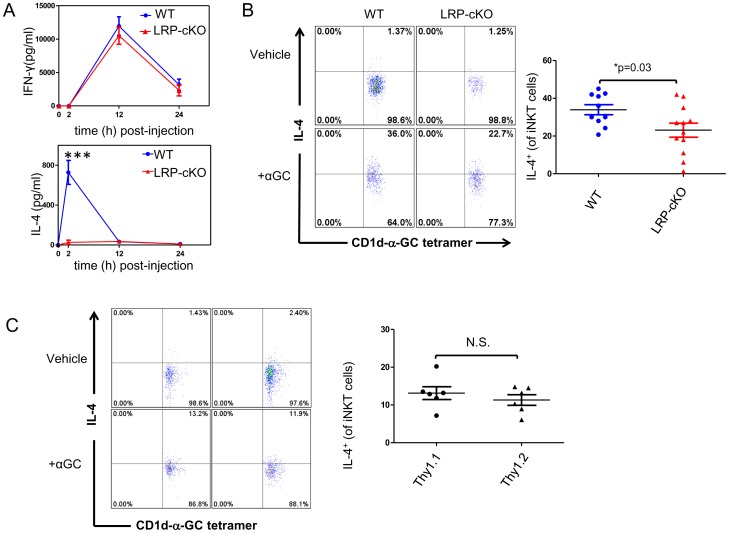
LRP deficiency in MΦ's results in normal IFN-γ response but decreased IL-4. **(A)** Mice were challenged with 4 µg αGC/mouse and serum assayed for IFN-γ and IL-4 by ELISA. Data points show standard error and mean. WT (n = 4 and 6 mice for 12 and 24 hour, respectively) and LRP-cKO (n = 5 and 4 for 12 and 24 hours, respectively), each point represents an individual mouse from a representative experiment of three separate experiments **(B)** WT (n = 7) and LRP-cKO mice (n = 8) challenged with 4 µg αGC/mouse or vehicle control. Intracellular cytokine stain was performed for IL-4 and antibodies for TCRβ, B220, and CD1d-αGC-tetramer to select iNKT cells. iNKT cells were defined as TCRβ^intermediate^, B220^-^, CD1d-αGC-tetramer^+^. Each point represents an individual mouse from a representative experiment of three separate experiments. **(C)** Chimeric mice were generated by transplanting Thy1.1 and LRP-cKO (Thy1.2) at a 1∶1 ratio into LysMCre^-/-^ LRP^flox/flox^ (Thy1.2) recipient mice. Isolation of splenic iNKT cells and intracellular cytokine stain of IL-4 was performed 4 weeks after transplantation. Dot plots on the left show percentage of Thy1.1^+^ or Thy1.2^+^ iNKT cells expressing IL-4. Graph on the right shows quantification of IL-4^+^ iNKT cells. Data presented from this experiment is the compilation of two separate experiments where each data point represents a single mouse. Bars show standard error and mean. *** denotes *p*<0.0001. *P*-value determined by Student's *t*-test.

Although we did not see an effect of MΦ-specific deletion of LRP on *in vitro* iNKT cell responses to αGC, we were interested in understanding whether the magnitude and/or kinetics of the *in vivo* iNKT cell response to αGC might be altered in LRP-cKO mice where the spatial integrity of the secondary lymphoid tissues would remain intact. To do this, we challenged WT and LRP-cKO mice with αGC (4 µg/mouse i.p.) and measured serum IFN-γ and IL-4 at 2, 12 and 24 hours. ELISA results from this experiment show the near absence of IL-4 production by iNKT cells in LRP-cKO mice (Figure 5A) However, the kinetics and levels of IFN-γ were not affected by LRP deficiency on MΦs).Lower doses of αGC (1 µg and 0.5 µg per mouse) did not recapitulate a similar difference between WT and LRP-cKO mice (Figure S6). In addition to this we measured a statistically significant reduction of IL-4^+^ iNKT cells (Figure 5B) and normal IFN-γ^+^ iNKT cells (Data not shown) in LRP-cKO cells by intracellular cytokine stain. The presence of normal IFN-γ responses is supported by similar levels of transactivation of T cells and NK cells as measured by CD69 upregulation (data not shown). These results suggest that, *in vivo,* MΦ LRP is not required for IFN-γ secretion, but can impact production of IL-4 by iNKT cells.

### Decreased IL-4 expression by iNKT cells in LRP-cKO mice is cell extrinsic

Although iNKT cells in LRP-cKO mice should not be directly affected we wanted to confirm that decreased IL-4 production in response to αGC was not due to an iNKT cell intrinsic deficiency. To do this we generated bone marrow chimeras using a 1∶1 ratio of bone marrow from Thy1.1 and LRP-cKO mice (Thy1.2) transplanted into recipient WT mice at a 1∶1 ratio. Four weeks after transplant, mice were injected with αGC and splenocytes analyzed for intracellular IL-4 cytokine stain. The results from this experiment (Figure 5C) show the production of IL-4 by Thy1.2^+^ iNKT cells (LRP-cKO) is rescued to Thy1.1^+^ iNKT cell levels in the 1∶1 mixed bone marrow chimeras. This result suggests that the decrease in IL-4 measured in LRP-cKO mice is cell extrinsic and that iNKT cells from LRP-cKO mice can respond normally in the presence of LRP-sufficient macrophages.

### LRP-cKO mice have a normal IgE response when challenged with αGC

**Figure 6:**
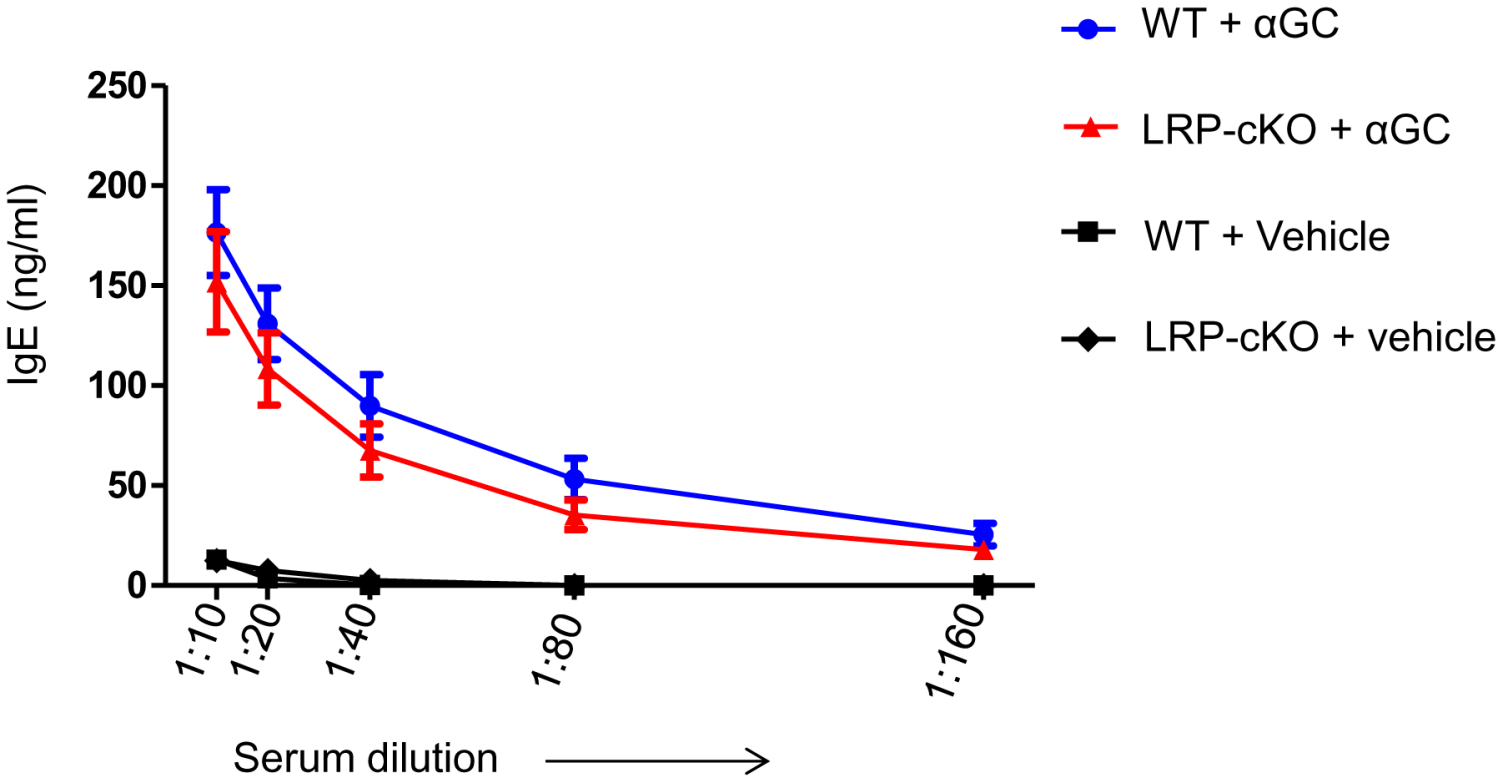
Induction of IgE in LRP-cKO mice is normal when compared to WT. Both WT (n = 8) and LRP-cKO (n = 6) mice were challenged with αGC (4 µg/mouse) and vehicle (WT, n = 3, LRP-cKO n = 2). Blood was collected at 0 and 6 days and total serum IgE levels measured by ELISA. Bars represent mean and standard error. These results are from one experiment.

To determine whether lack of IL-4 response in LRP-cKO mice to αGC could lead to a deficiency in the adaptive immune response, we measured production of IgE. It has been established that a single dose of αGC can lead to a robust increase in the presence of serum IgE 6 days after αGC injection[47]. To test this, we injected WT and LRP-cKO mice and obtained serum at day 6 and measured IgE levels by ELISA (Figure 6). Titration curves of IgE in this experiment show that, despite a lack of early burst of IL-4, LRP-cKO mice are still able to produce a normal, although reduced, IgE response. This result suggests that, while LRP deficiency in macrophages decreases iNKT cell IL-4 production, the main cytokine involved in IgE production, it is not necessary for increased IgE.

## Discussion

Several studies have reported that activation of the immune system can reciprocally regulate the expression of lipoprotein receptors in APCs. For example, exposure of macrophages to IFN-γ can exert dramatic changes of LRP [48], [49], very low density lipoprotein (VLDL) receptor [50], and scavenger receptors (SRs)[51]. Far less however, is known about how these changes in lipid-binding receptors might influence immune responses. LRP's ubiquitous nature, ligand diversity and involvement in intracellular signaling cascades allow it to be a receptor capable of affecting a multitude of cellular processes. Unfortunately, the examination of LRP's expression and function has been largely confined to human cell lines [52], [53], mouse cell lines [54], [55] and primary mouse cells [26], [37]. The LRP^flox/flox^ mouse model [38] has allowed a wide number of conditional knockouts which verify its multicellular distribution and function [29], [56], [57]. By crossing it with LyzM-Cre mice, we observed close to a 70% reduction of LRP in primary cells (Figure 3), which is reminiscent to other Cre^+/-^ models such as the adipocyte specific aP2-Cre and neuron specific synapsin-Cre [58]. In all these cases a full deletion was not observed, but rather a near complete reduction of LRP. In this study, we show that in the steady state, T cells express low levels of LRP followed by B cells. DCs express a small amount of LRP, but MΦs express higher levels of LRP (Figure 1). This demonstrates that on immune cells, LRP expression is largely restricted to phagocytes and professional APCs supporting its role in antigen presentation and modulation of immunity.

Using a conditional mouse model of MΦ LRP knockout, we show that reduction of LRP expression in MΦs does not influence iNKT activation *in vitro* (Figure 4C and Figure S5), but abolishes IL-4 production *in vivo* (Figure 5). This lack of IL-4 response was not the result of altered development and/or homeostasis of iNKT cells (Figure S3). Because we did not observe changes in iNKT cell activation using single cell suspensions *in vitro*, but did see a near absence of early iNKT cell IL-4 production following *in vivo* challenge, we hypothesize that spatial orientation of the LRP expressing macrophages may be important to early iNKT cell responses. We focused our studies on spleen because 1) marginal zone macrophages express receptors that allow them to capture blood borne antigens ([59], 2) spleen contains an elaborate distribution network which disseminates molecules present in the blood to white pulp where APCs are located [60], and 3) iNKT cells that reside in the spleen are exposed to blood borne antigen within minutes after they enter the bloodstream [43]. Our attempts to measure iNKT cell activation in liver and LN shortly after αGC injection were not successful. Therefore, whether iNKT cells in liver and lymph nodes undergo a similar exposure to blood borne antigens is an issue that needs to be addressed in future studies.

Indeed, previous studies have demonstrated that MΦs in the splenic marginal zone (MZ) are the first APCs to activate iNKT cells in response to blood-borne glycolipids [43]. Using time-lapse multiphoton microscopy the authors showed that, following antigen challenge, splenic iNKT cells quickly localize to the MZ and come into contact with MZ CD169^+^ MΦs. Interestingly, when the MZ integrity was compromised following injection of clodronate liposomes, a method of MΦ-specific depletion, there was a dramatic decrease in iNKT cell cytokine production in response to *in vivo* challenge with particulate αGC [41]. *In vitro* comparison of antigen presenting efficiency of DCs and MZ MΦs to iNKT cells showed that, although MZ MΦs were able to activate iNKT cells *in vitro*, DCs were clearly superior. Therefore, the data suggest that the spatial interaction of iNKT cells with the MZ MΦs soon after injection of αGC is the most plausible explanation for the observed *in vivo* decreases in iNKT cell activation.

More specifically, it is possible that at 2 hours the localization of LRP-expressing MΦs in the MZ induces iNKT cells to secrete IL-4. However, at later times other APCs (such as DCs) are primarily responsible for iNKT cell activation and the resulting IFN-γ response. Because *in vitro* disruption of the spleen to make single cell suspensions would destroy this spatial integrity allowing for iNKT cells to be activated by DCs, this decrease in IL-4 production in response to αGC would not be observed. In support of this hypothesis, our study demonstrates that splenic CD169^+^ MZ MΦs express higher levels of LRP than the CD169^-^ MΦs (Figure 2). Therefore LRP expression in these CD169^+^ MΦs may influence the early activation of iNKT cells and consequently lead to decreased IL-4 at 2 hours.

The expression of surface receptors that can direct glycolipid uptake and subsequent iNKT cell activation suggests multiple pathways are responsible for delivery to APCs. To date, no specific receptor has been described that carries out this function. In fact, the hydrophobic nature of glycolipids prevents their individual presence in the serum and would require their association to serum-borne particles. Van Den Elzen *et al* found that apoE-containing lipoproteins, specifically VLDL could associate with iNKT cell activating glycolipids such as αGC's close family member galactosyl(α1-2) galactosyl ceramide [14]. The investigators went on to demonstrate that apoE-associated glycolipid was efficiently taken up by the LDLr and enhanced iNKT cell activation. More recent studies demonstrate that, like the LDLr, other lipid receptors such as scavenger receptor (SRA) can bind and facilitate uptake of glycolipid antigens by DCs to activate iNKT cells [15]. Preference for receptor/glycolipid interaction was determined to be more dependent on chemical structure of the lipid. Interestingly, both SRA- and LDLr-deficient DCs showed decreased ability to activate the iNKT cell hybridoma DN32.D3 in response to αGC *in vitro*. However, *in vivo* challenge of SRA and LDLr-deficient mice with soluble αGC led to the same blunted IL-4 response we observed in LRP-cKO mice (Figure 5). How these receptors contribute to such different cytokine responses remains to be clarified. One possibility is that all three receptors (LRP, SRA and LDLr) are required for an optimal IL-4 response *in vivo*. Another may be that loss of any one of these receptors leads to changes in intracellular lipid compositions and that the iNKT cell IL-4 response is sensitive to these changes. We did not observe changes in uptake of BODIPY-αGC in LRP-cKO MΦs in our studies (Figure S4A-B), However, it is possible that this is the result of the limitation of our *in vitro* system. Surprisingly, in a pulse chase experiment, we did see increased appearance of BODIPY-αGC in LRP-cKO MΦ indicating that lipid exchange may be accelerated in the absence of LRP (Figure S4C and S4D). Therefore, one might hypothesize that more rapid replacement of αGC with endogenous lipids in LRP-cKO MΦs results in a blunted, early IL-4 response *in vivo*. Further investigation is warranted to determine whether there are *in vivo* changes in antigen-uptake or whether loss of these receptors affects lipid rafts, or if other lipid pools important for cell activation are required.

Collectively, this study demonstrates that LRP expression in MΦs does not alter total activation of iNKT cells *in vivo* but, like the LDLr and SRA, LRP plays a role in IL-4 secretion by iNKT cells. This result is remarkable as it suggests a scenario where LRP can be manipulated to modulate iNKT cell responses. Activation of iNKT cells has been associated with altered progression of cancer, autoimmune disorders and atherosclerosis. Understanding how lipoprotein receptors affect their function can lead to targeted therapies to harness the immunoregulatory potential of iNKT cells.

## Supporting Information

Figure S1 **CD1d expression in spleen MΦs, liver MΦs and double positive (CD4^+^ CD8^+^) thymocytes**. (A) Dot plots (left panels) of pMΦs stained with fluorochrome-conjugated antibodies B cells (B220), and pMΦs (CD11b). Representative histogram (middle) shows LRP expression gated on CD11b^+^ cells. Mean fluorescent intensity (LRP antibody – isotype antibody). (B) ELISA measurement of IL-2 production by DN32.D3 hybridomas cocultured with WT or LRP-cKO thymocytes. Data points show standard error and mean. Data is representative of three independent experiments.
(TIF)

Figure S2 **Thymic analysis of iNKT cells.** Representative dot plots (left panels) of thymic cells and quantification (scatter plots). Cells were stained with fluorochrome conjugated antibodies for B cells (B220), T cells (TCRβ), αGC-CD1d-tetramer, and homeostatic markers of iNKT cells: NK1.1, CD4, CD28 and CD40L.
(TIF)

Figure S3 **LRP-cKO deficiency in MΦs leads to normal development of iNKT cells.** **(A)** Spleen and **(B)** liver mononuclear cells from 8-to-12 week old WT and LRP-cKO were stained with fluorochrome-conjugated antibodies for T cells (TCRβ), B cells (B220) and CD1d-αGC tetramer. iNKT gate (TCRβ ^int^Tet^+^B220^-^) shows frequency of iNKT cells. Graphs show quantification of total iNKT cell number and frequency in **(B)** spleen and **(C)** liver, bars represent mean and standard error. Spleen (left dot plot) and liver (right dot plot) iNKT cells stained with **(D)** PD-1 and **(E)** Ly-49 fluorochrome conjugated antibodies. Shown are representative histograms gated on iNKT cells. Results are representative of one experiment from three independent experiments.
(TIF)

Figure S4 **Peritoneal MΦs uptake of fluorescently labeled αGC (BODIPY-αGC).** pMΦs from WT (n = 3) and LRP-cKO (n = 4) were incubated with 1 mg/ml of BODIPY-αGC and harvested at the indicated times. This was followed by incubation with fluorochrome conjugated antibodies for CD11b, CD11c and B220. Shown are **(A)** representative histograms and **(B)** MFI quantification in CD11b^+^CD11c^-^B220^-^ pMΦs pulsed with unlabeled αGC for four hours and chased with labeled BODIPY-αGC at the indicated hours. BODIPY-αGC (MFI) was measured by flow cytometry MΦs. **(C)** Representative histograms of WT (n = 3) and LRP-cKO (n = 3) and **(D)** MFI quantification in CD11b^+^CD11c^-^B220^-^ pMΦs. Results shown are representative of an experiment from three independent experiments. Bars represent mean and standard error and * denotes p<0.05.
(TIF)

Figure S5 **WT and LRP-cKO splenocytes challenged with αGC.** Splenocytes from WT and LRP-cKO mice were stimulated with indicated concentration of αGC for 24, 48 and 72 hours. Supernatants were assayed for IFN-γ and IL-4 by ELISA. Results are from one representative experiment of three independent experiments. Data points show standard error and mean of 3 mice in each group. N.S. stands for data sets that are statistically not significant.
(TIF)

Figure S6 **WT and LRP-cKO mice challenged with αGC.** Mice were challenged with 1 µg and 0.5 µg αGC/mouse and blood collected at the indicated time points. Serum was assayed for IFN-γ and IL-4 by ELISA. Data points show standard error and mean. WT (n = 3 for 2, 12 and 24 hours, respectively) and LRP-cKO (n = 3 for 12 and 24 hours). Results shown are representative of 3 independent experiments.
(TIF)
