# Supplementary figures and images for: Specific Deletion of LDL Receptor-Related Protein on Macrophages Has Skewed In Vivo Effects on Cytokine Production by Invariant Natural Killer T Cells
Source: PLoS One. 2014 Jul 22;9(7):e102236. doi: 10.1371/journal.pone.0102236 (PMC4106787; doi:10.1371/journal.pone.0102236)

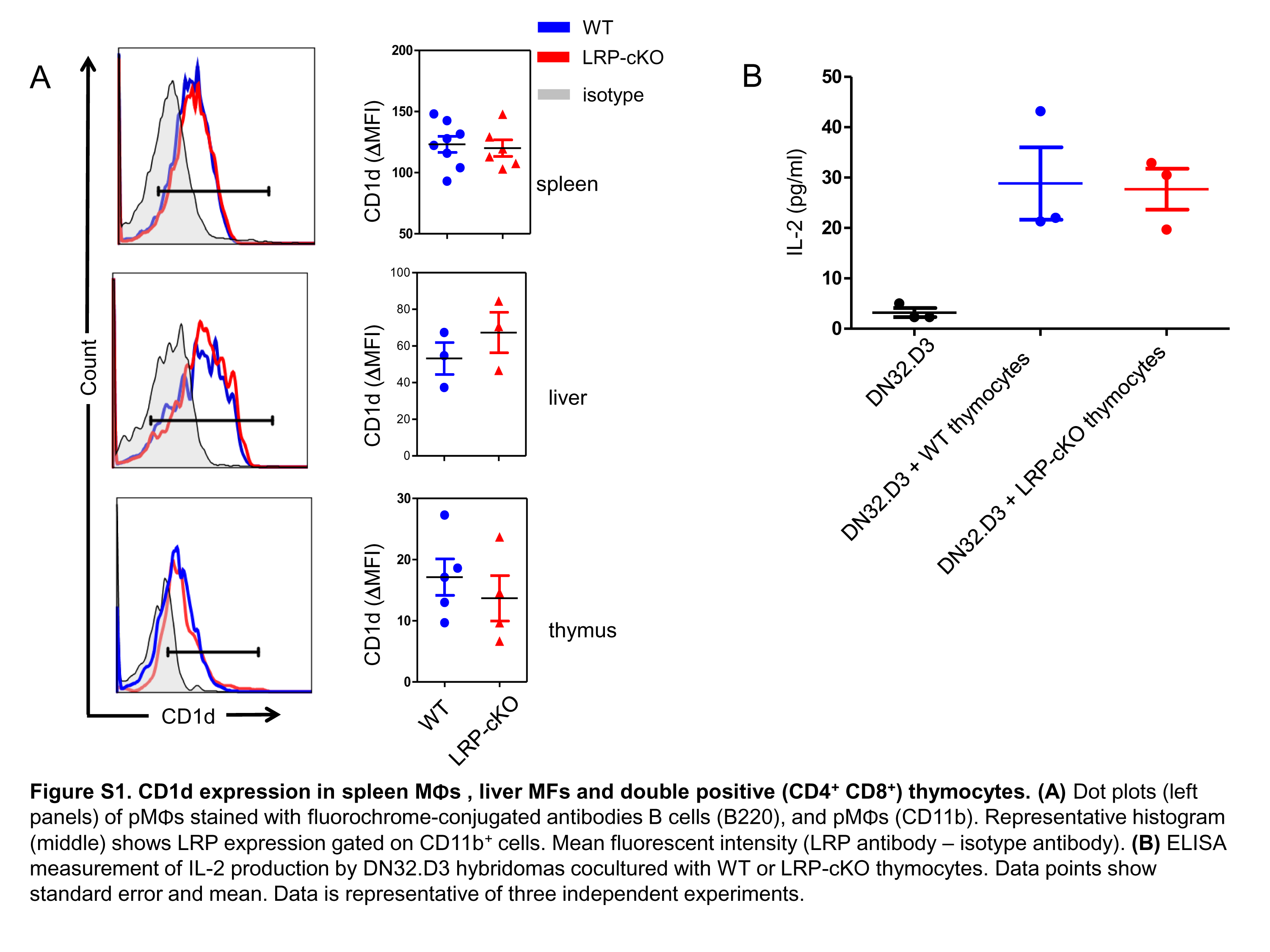

Supplement: Figure S1 — CD1d expression in spleen MΦs, liver MΦs and double positive (CD4+ CD8+) thymocytes. (A) Dot plots (left panels) of pMΦs stained with fluorochrome-conjugated antibodies B cells (B220), and pMΦs (CD11b). Representative histogram (middle) shows LRP expression gated on CD11b+ cells. Mean fluorescent intensity (LRP antibody – isotype antibody). (B) ELISA measurement of IL-2 production by DN32.D3 hybridomas cocultured with WT or LRP-cKO thymocytes. Data points show standard error and mean. Data is representative of three independent experiments. (TIF) [file pone.0102236.s001.tif]

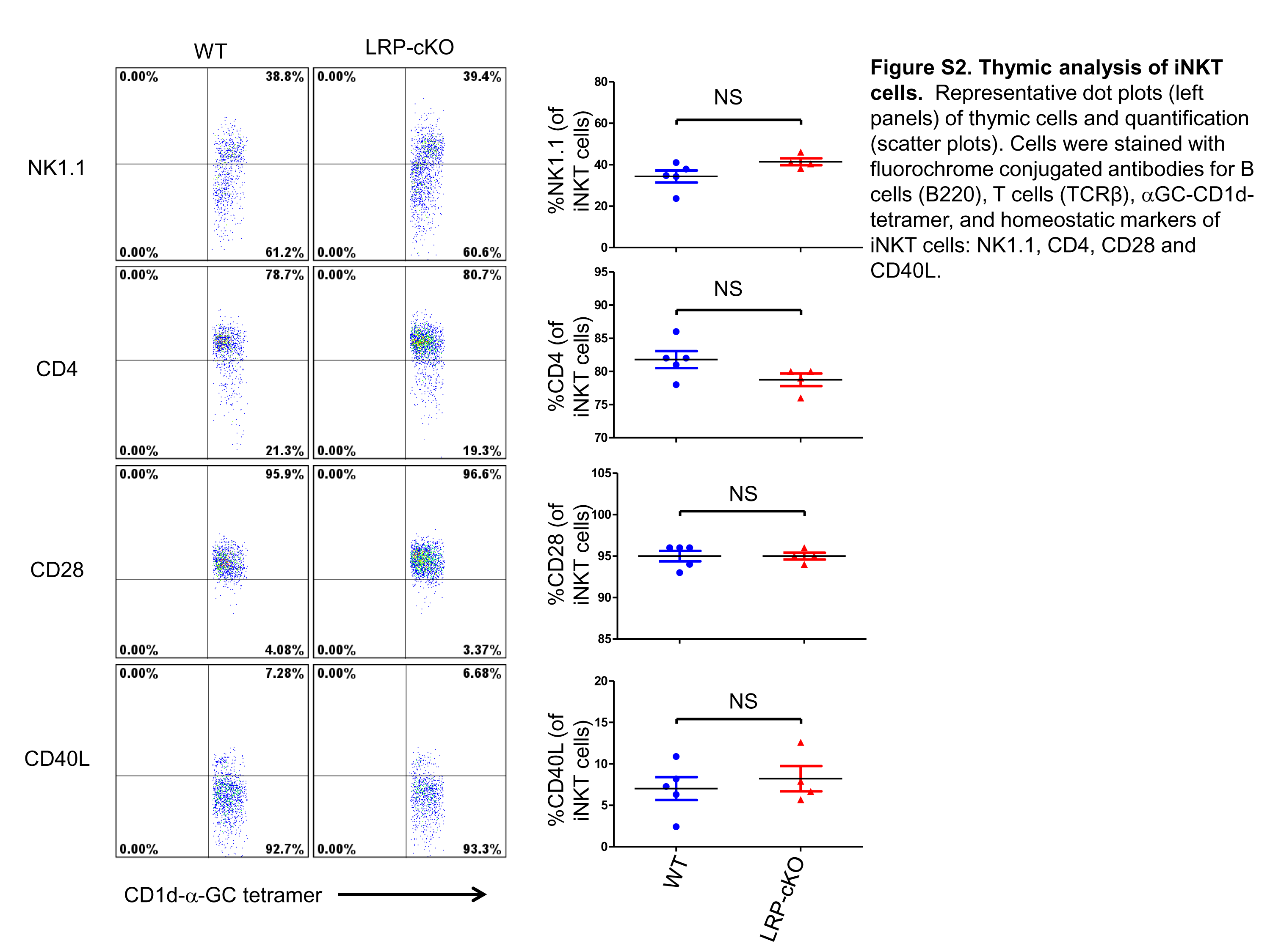

Supplement: Figure S2 — Thymic analysis of iNKT cells. Representative dot plots (left panels) of thymic cells and quantification (scatter plots). Cells were stained with fluorochrome conjugated antibodies for B cells (B220), T cells (TCRβ), αGC-CD1d-tetramer, and homeostatic markers of iNKT cells: NK1.1, CD4, CD28 and CD40L. (TIF) [file pone.0102236.s002.tif]

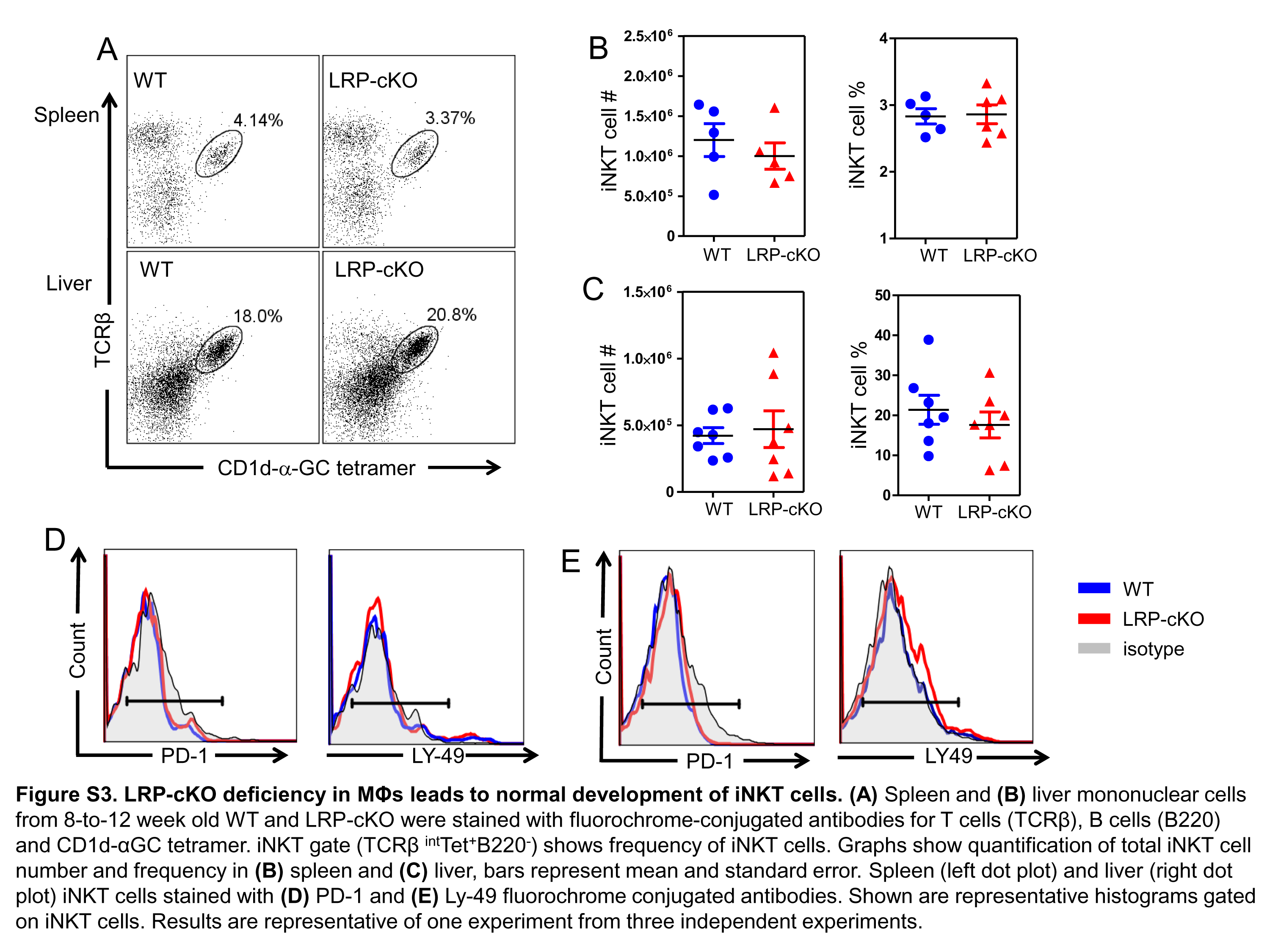

Supplement: Figure S3 — LRP-cKO deficiency in MΦs leads to normal development of iNKT cells. (A) Spleen and (B) liver mononuclear cells from 8-to-12 week old WT and LRP-cKO were stained with fluorochrome-conjugated antibodies for T cells (TCRβ), B cells (B220) and CD1d-αGC tetramer. iNKT gate (TCRβ intTet+B220-) shows frequency of iNKT cells. Graphs show quantification of total iNKT cell number and frequency in (B) spleen and (C) liver, bars represent mean and standard error. Spleen (left dot plot) and liver (right dot plot) iNKT cells stained with (D) PD-1 and (E) Ly-49 fluorochrome conjugated antibodies. Shown are representative histograms gated on iNKT cells. Results are representative of one experiment from three independent experiments. (TIF) [file pone.0102236.s003.tif]

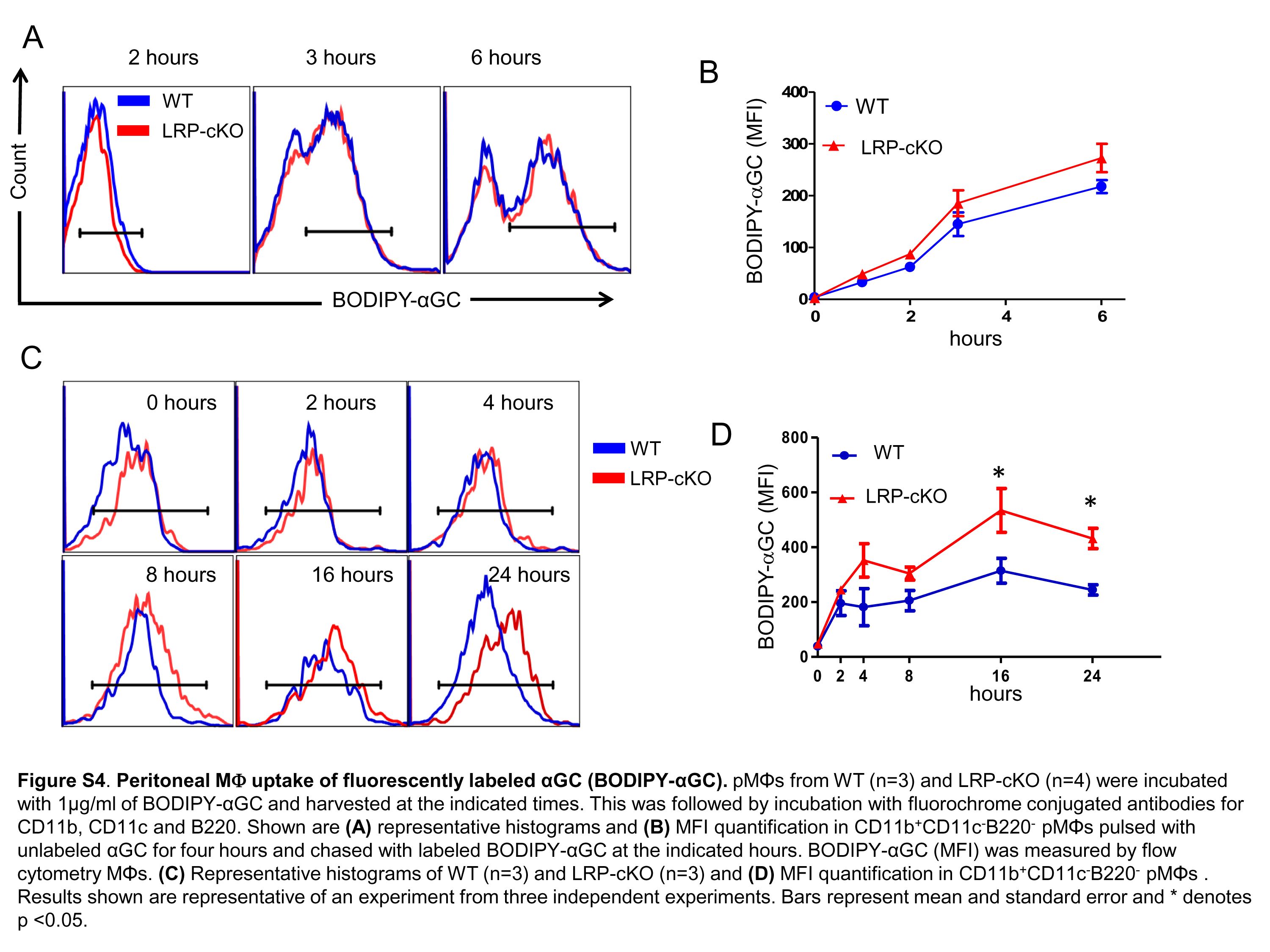

Supplement: Figure S4 — Peritoneal MΦs uptake of fluorescently labeled αGC (BODIPY-αGC). pMΦs from WT (n = 3) and LRP-cKO (n = 4) were incubated with 1 mg/ml of BODIPY-αGC and harvested at the indicated times. This was followed by incubation with fluorochrome conjugated antibodies for CD11b, CD11c and B220. Shown are (A) representative histograms and (B) MFI quantification in CD11b+CD11c-B220- pMΦs pulsed with unlabeled αGC for four hours and chased with labeled BODIPY-αGC at the indicated hours. BODIPY-αGC (MFI) was measured by flow cytometry MΦs. (C) Representative histograms of WT (n = 3) and LRP-cKO (n = 3) and (D) MFI quantification in CD11b+CD11c-B220- pMΦs. Results shown are representative of an experiment from three independent experiments. Bars represent mean and standard error and * denotes p<0.05. (TIF) [file pone.0102236.s004.tif]

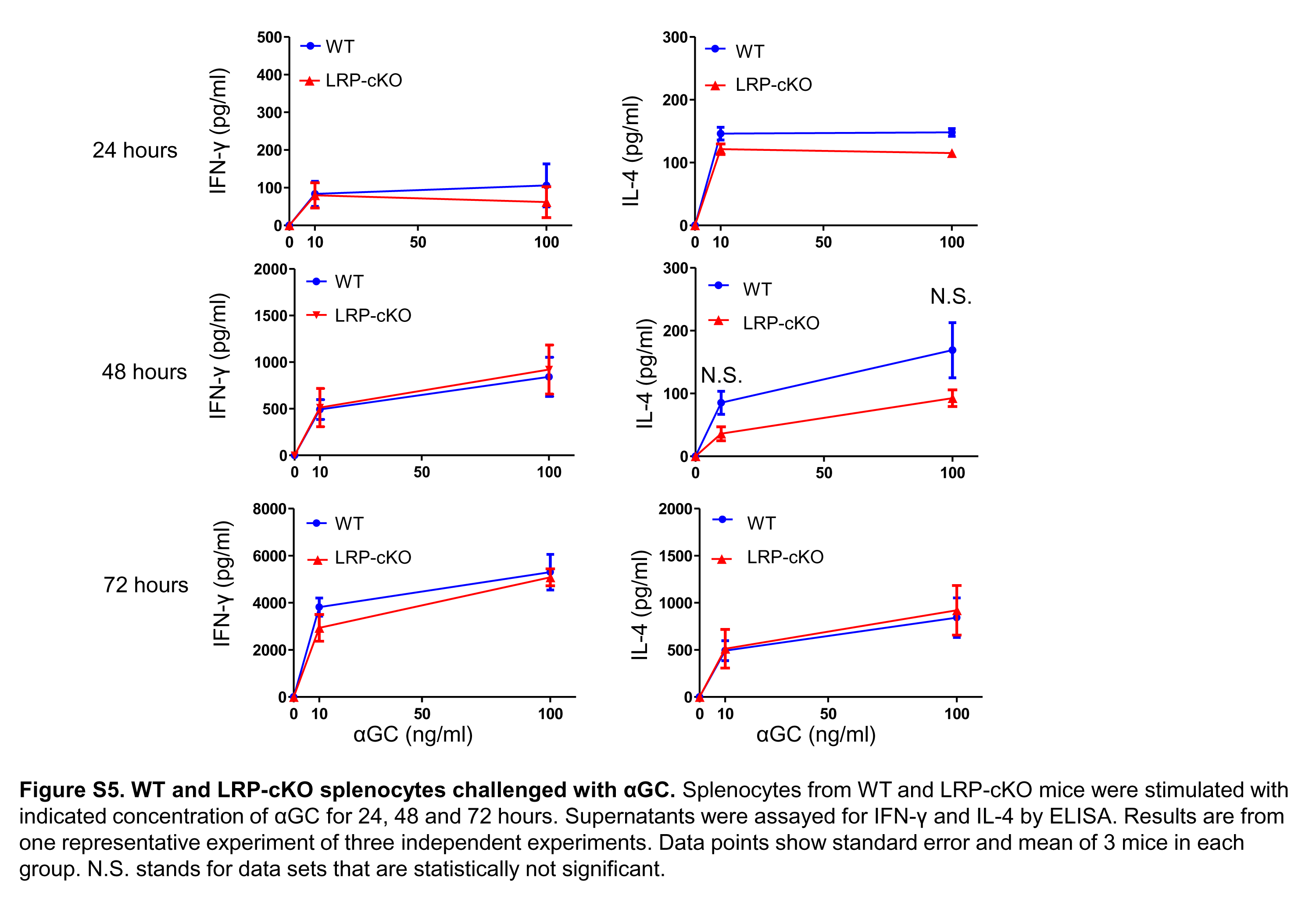

Supplement: Figure S5 — WT and LRP-cKO splenocytes challenged with αGC. Splenocytes from WT and LRP-cKO mice were stimulated with indicated concentration of αGC for 24, 48 and 72 hours. Supernatants were assayed for IFN-γ and IL-4 by ELISA. Results are from one representative experiment of three independent experiments. Data points show standard error and mean of 3 mice in each group. N.S. stands for data sets that are statistically not significant. (TIF) [file pone.0102236.s005.tif]

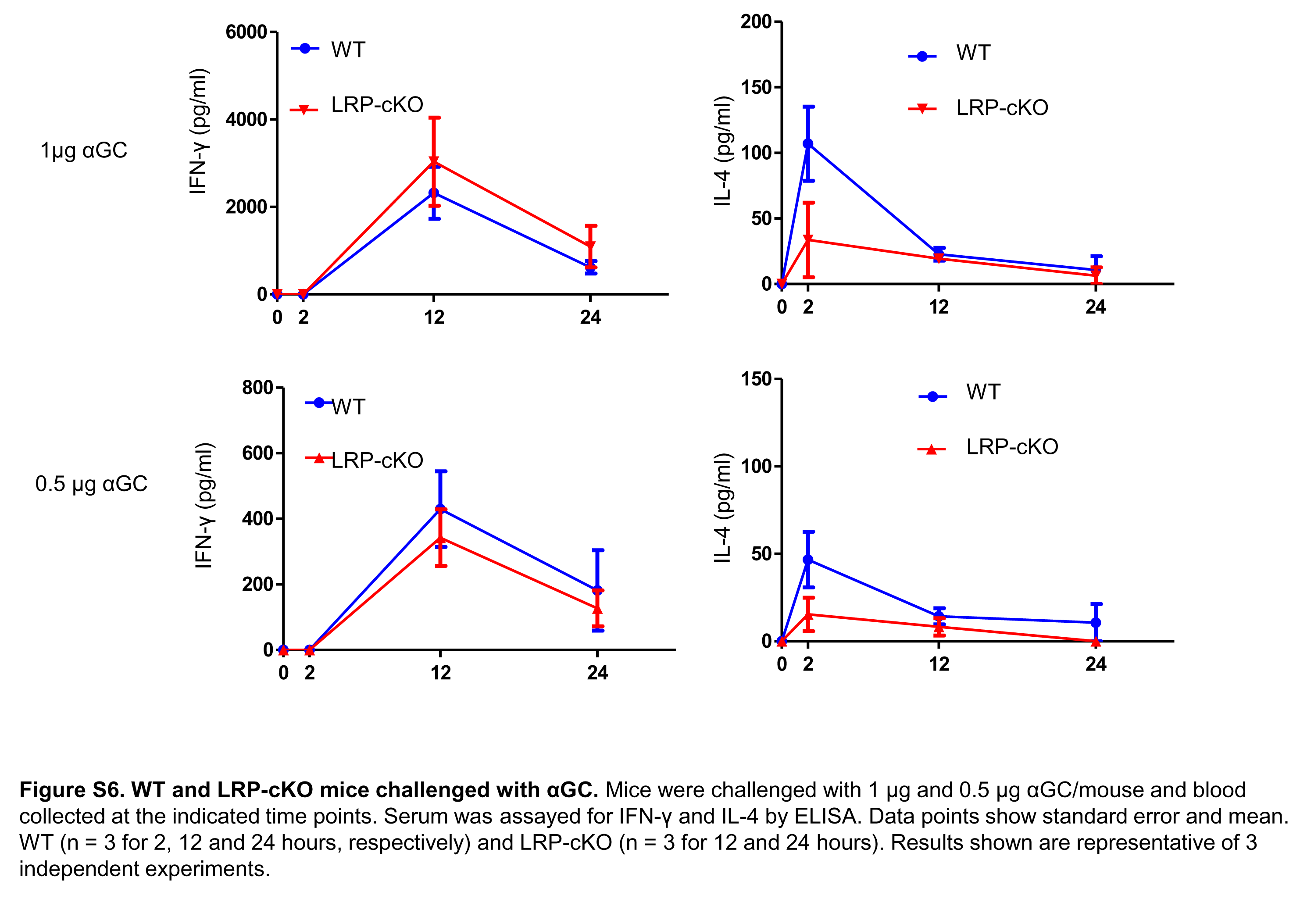

Supplement: Figure S6 — WT and LRP-cKO mice challenged with αGC. Mice were challenged with 1 µg and 0.5 µg αGC/mouse and blood collected at the indicated time points. Serum was assayed for IFN-γ and IL-4 by ELISA. Data points show standard error and mean. WT (n = 3 for 2, 12 and 24 hours, respectively) and LRP-cKO (n = 3 for 12 and 24 hours). Results shown are representative of 3 independent experiments. (TIF) [file pone.0102236.s006.tif]
